# Heritability and Transcriptional Impact of JAK3, STAT5A and STAT6 Variants in a Tyrolean Family

**DOI:** 10.3390/ijms27020913

**Published:** 2026-01-16

**Authors:** Hye Kyung Lee, Teemu Haikarainen, Yasemin Caf, Priscilla A. Furth, Ludwig Knabl, Olli Silvennoinen, Lothar Hennighausen

**Affiliations:** 1 National Institute of Diabetes, Digestive and Kidney Diseases, National Institutes of Health, Bethesda, MD 20892, USA; priscilla.furth@nih.gov; 2 Faculty of Medicine and Health Technology, Tampere University, 33100 Tampere, Finland; teemu.haikarainen@tuni.fi (T.H.); olli.j.silvennoinen@helsinki.fi (O.S.); 3 Fimlab Laboratories, 33900 Tampere, Finland; 4 Y2L2Science GmbH, Hauptplatz 4, 6511 Zams, Austria; yasemin.caf@y2l2science.com (Y.C.); ludwig.knabl@y2l2science.com (L.K.); 5 Institute of Biotechnology, Helsinki Institute of Life Science (HiLiFE), University of Helsinki, 00014 Helsinki, Finland

**Keywords:** JAK-STAT signaling pathway, germline genetic variants, immune transcriptome profiling, variant penetrance and heritability

## Abstract

The Janus Kinase (JAK) and Signal Transducers and Activators of Transcription (STAT) pathways regulate a range of biological processes, including immune response and hematopoiesis. While a major research focus has been on somatic human mutations in disease, less is known about the heritability of germline variants and their physiological impact. This study addresses an important issue in population genetics: the context-dependent effects and incomplete penetrance of rare genetic variants in immune pathways. Here we identify the rare JAK3^P151R^, JAK3^R925S^, STAT5A^V494L^, and STAT6^Q633H^ variants in an extended family spanning three generations, integrate in silico analyses and AlphaFold 3 structural predictions, and investigate the immune transcriptomes in probands carrying one or more variants. All four variants are inherited through the germline without any evident clinical or physiological manifestations in the carriers. As individual variants, not all persons carrying a specific variant showed the same immune transcriptome. The presence of activated basal transcriptomes was limited to some, but not all, individuals carrying the above variants. A next step in understanding the role of germline variants will be to understand how and why other factors, including both other germline variants and environmental and developmental factors, influence the likelihood of expression of an activated basal transcriptome.

## 1. Introduction

Janus Kinases (JAKs) and Signal Transducers and Activators of Transcription (STATs) play indispensable roles in translating cytokine signals into transcriptional responses that regulate a range of biological processes, including body growth [1], lactation [2], hematopoiesis [2,3,4], inflammation, and antiviral defense. Dysregulated JAK/STAT signaling has been implicated in chronic inflammation and autoimmune disease, and somatic variants have been identified predominantly in hematological malignancies. These include the classical JAK2^V617F^ variant in myeloproliferative neoplasms (MPNs) [5,6] and Src Homology 2 (SH2) domain variants of STAT family members [7,8].

We recently identified a range of germline JAK/STAT variants and linked some to an exacerbated innate immune response after vaccination [9,10] and an elevated baseline immune transcriptome [11]. Access to community-based cohorts permitted us to address yet unanswered questions focused on the presence of JAK/STAT variants in large families. First, it is not well understood to what extent these variants are transmitted through the germline, especially in individuals carrying two or more variants. Second, the combinatorial transcriptional impact of several variants in individuals remains to be understood. Here we identified a large family carrying two JAK3 variants (JAK3^P151R^ and JAK3^R925S^), as well as STAT5A^V494L^ and STAT6^Q633H^ variants. These germline variants are very rare but no definitive biological information has been reported in the literature [12]. The JAK3^P152R^ variant results in a non-conservative amino acid change in the FERM domain and the JAK3^R925S^ variant is also a non-conservative amino acid change in the kinase domain. STAT5A^V494L^, a novel germline variant, represents a conservative substitution, located in a solvent-exposed loop at the end of the DNA binding domain of the protein. The STAT6^Q633H^ variant is located in the SH2 linker region and gain.

The novel conceptual contribution of our study extends beyond the report-of-function (GOF) germline mutations have been identified in this region [13]. We used in silico analyses and AlphaFold 3 (AF3) protein structure predictions to assess the pathogenicity of these variants and gauged their impact in individuals carrying one or more variants.ing of rare JAK/STAT germline variants in a large family and their inheritance through three generations to understanding how these variants could shape the immune transcriptome. While this concept is tenable, the low frequency of these variants makes it difficult, if not impossible, to identify additional individuals displaying these variant combinations. This highlights the possibility that combinations of JAK/STAT variants have an individual impact. However, this complexity is further expanded by including variants in Tyrosine Kinase 2 (TYK2) and additional proteins acting upstream of JAK/STAT, such as receptors, and downstream, such as SOCS proteins. Just by including TYK2 variants, each of the investigated family members has a unique combination of variants. Lastly, while AF3 is a powerful tool in protein modeling, it might not provide sufficient depth to identify subtle changes leading to protein dimerization, receptor–JAK-STAT interactions, STAT nuclear translocation, and transcriptional activation. Structural neutrality, as shown in some of our variants, does not necessarily equate to a lack of biological impact.

## 2. Results

In a quest to understand the heritability of JAK/STAT variants and further explore their combined transcriptional impact, we searched for families carrying two or more variants in any of the three JAK and seven STAT proteins. Here we report on a family we had recruited during the COVID-19 pandemic to investigate the innate immune response after COVID-19 Omicron infection [14]. We chose this family for a more detailed investigation of the influence of common and rare variants in the JAK/STAT signaling pathway on the immune transcriptome. Importantly, members of three generations of this family were examined; all family members lived in the same geographical area and had similar lifestyles. This enabled us to make more precise statements about the significance of the rare variants we found, as environmental or lifestyle-associated confounders could be largely ruled out. Furthermore, a particularly rare variant (STAT6^Q633H^) occurred in members of all three generations, allowing us to assess its potential pathogenicity across different stages of life. We obtained extensive medical data and immune transcriptome data from this family, which was obtained when examining family members during an Omicron infection that affected almost all members at the same time.

**Figure 1:**
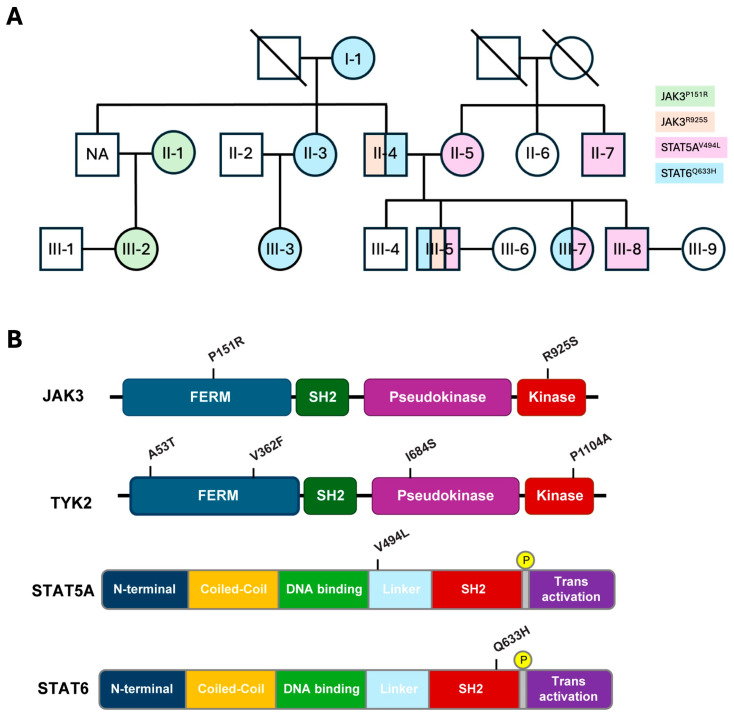
Identification of Janus Kinase (JAK)/Signal Transducers and Activators of Transcription (STAT) variants in an extended Tyrolean family. (**A**) Family tree. (**B**) Domain structures of JAK3, STAT5A, and STAT6 and location of the identified variants. JAK3^P151R^ is located in the FERM domain, JAK3^R925S^ in the kinase domain, and STAT5A^V494L^ in the linker between the DNA-binding domain (DBD) and the Src Homology 2 (SH2) domain. STAT6^Q633H^ is located in the SH2 domain.

Both RNA-seq and whole exome sequencing (WES) data collected from 17 members of a Tyrolean family spanning three generations revealed two *JAK3* variants, and one variant each in *STAT5A* and *STAT6* was identified (Figure 1). The JAK3^P151R^ and JAK3^R925S^ variants are in the FERM domain and the kinase domain, respectively. The STAT5A^V494L^ variant is within the linker separating the DNA-binding domain (DBD) and the SH2 domain, and the STAT6^Q633H^ variant is positioned at the C-terminal boundary of the SH2 domain. The STAT6^Q633H^ variant was identified in six individuals in three generations and the STAT5A^V494L^, JAK3^P151R^, and JAK3^R925S^ variants in two generations. Two individuals carried only a single variant, one person carried the STAT6^Q633H^ and JAK3^R925S^ variants, one carried the STAT6^Q633H^ and STAT5A^V494L^ variants, and one person carried three variants, STAT6^Q633H^, STAT5A^V494L^, and JAK3^R925S^ (Table S1). Both JAK3^P151R^ and JAK3^R925S^ are rare variants; STAT5A^V494L^ has not been listed in databases, and STAT6^Q633H^ is an ultra-rare variant with an allele frequency of approximately 10^−4^ in gnomAD and All of Us (Table 1). These findings demonstrate that all four variants are inherited through the germline without any evident clinical or physiological manifestations in the carriers. Importantly, individual II-4 passed on the STAT6^Q633H^ and JAK3^R925S^ variants to his son who also inherited the STAT5A^V494L^ variant from his mother, II-5 (Figure 1). Ten of the eleven family members with *JAK*/*STAT* variants also featured *TYK2* variants (Table S1). Three family members carried only *TYK2* variants, and three family members did not carry any *JAK*/*STAT*/*TYK2* mutations. Coinheritance of *JAK*/*STAT* and *TYK2* variants has also been described in other cohorts in South Korea [10] and Austria [9].

To evaluate the potential pathogenicity of these four *JAK*/*STAT* variants and the four *TYK2* variants, we employed the NIH ClinVar database and additional in silico prediction tools (Table 1). While no ClinVar records were available for STAT6^Q633H^, STAT5A^V494L^ and JAK3^R925S^, JAK3^P151R^ was considered benign. AlphaMissense [15], a state-of-the-art computational tool, predicted an ambiguous score for JAK3^R925S^ and benign scores for the other three variants. The PolyPhen-2 [16] scores predicted a significant impact of the JAK3^R925S^ and STAT6^Q633H^ variants. The REVEL (Rare Exome Variant Ensemble Learner) [17] scores suggested a higher probability of pathogenicity for JAK3^R925S^, STAT5A^V494L^, and STAT6^Q633H^. While the *TYK2* variants V362F, I684S, and P1104A are quite common, with an AF between approximately 2 × 10^−1^ and 2 × 10^−2^, the A53T variant has an AF of 7 × 10^−3^. All four are considered benign or likely benign in ClinVar. AlphaMissense and PolyPhen2 categorizes TYK2^I684S^ and TYK2^P1104A^ as pathogenic. TYK2^P1104A^ has also been described as an autoimmune protective variant [18] and suppressor of inflammation [19]. Similarly, TYK2^I684S^ has been reported to protect from psoriasis and impair memory T-cells [20]. To assess potential epistatic effects of the *JAK*/*STAT* and *TYK2* variants identified in this family, we performed epistasis analyses using the Epistasis Disease Atlas [21] and detected no significant interactions between these variants and other immune-related genes. Maximum-likelihood model scores from NeEDL are summarized in Table S2.

### 2.1. Structural Analysis of JAK and STAT Mutations

JAK3 has very little structural information available compared to other family members, and only the structure of the JAK3 kinase domain has been experimentally resolved. Therefore, AI-based structure prediction with AF3 was used to obtain structural information of the two identified mutations. Notably, structural prediction of JAK3 with AF3 results in a highly similar tertiary structure when compared to JAK1, JAK2, and TYK2 [18]. We also analyzed the mutations in the context of a cryogenic electron microscopy (cryo-EM) structure of activated JAK dimers (PDB code 8EWY), but in the activated conformation, both mutated residues faced the solvent and did not participate in intra-domain or JAK-JAK interactions across the dimer interface. Therefore, it is likely that the mutations exert their effects, if any, on the monomeric, autoinhibited conformation.

**Figure 2:**
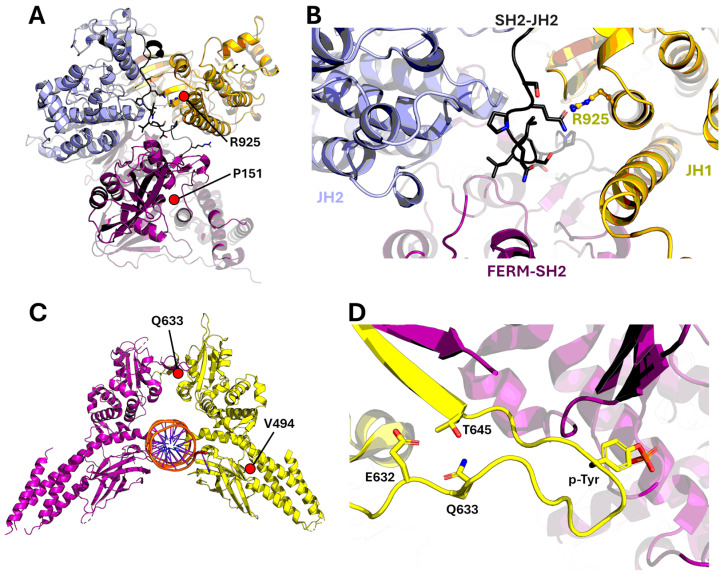
Structural analysis of the mutants. (**A**) The location of Pro125 and Arg925 in the AF3 model. Orchid: FERM-SH2 domains; Purple: JH2 domain (Pseudokinase domain); Yellow: JH1 domain (Kinase domain); Black: SH2-JH2 linker. (**B**) The arginine side chain points towards the SH2-JH2 linker. FERM-SH2 is colored in purple, JH2 in light blue, JH1 in yellow, and the SH2-JH2 linker in dark gray. (**C**) STAT6 structure (PDB code 4Y5W) [24] showing the location of STAT5A V494L and STAT6 Q633H mutations. Orchid: STAT6 protein; Yellow: STAT5A protein. (**D**) Close-up view of the STAT6 Q633H mutation next to the phosphotyrosine (p-Tyr) binding site.

The P151R mutation is located in the FERM-SH2 domain in a short linker between two a-helices (Figure 2A). The residue is solvent-exposed, and based on the structural analysis, the mutation would not disrupt or create any specific interactions. However, due to its rigid cyclic structure, introducing a more flexible residue in the place of proline could lead to disruption of the linker and the conformation of the protein fold, at least locally. The R925S mutation is positioned in the C-lobe of the kinase domain, and in the AF3 model, the arginine side chain faces the SH2-JH2 linker, which runs in the interface of FERM-SH2, pseudokinase, and kinase domains (Figure 2A,B). The mutation could disrupt the linker conformation and thus the autoinhibited conformation of JAK3. Importantly, the SH2-JH2 linker is a hotspot of pathogenic JAK mutation and harbors activating mutations, especially in hematologic malignancies, e.g., JAK3^M511I^ [22] and JAK2^K539L^ [23].

The identified STAT5A^V494L^ mutation is located in a solvent-exposed loop at the end of the DNA binding domain (Figure 2C). Wild-type valine does not participate in any direct interactions via its side chain, which points towards the solvent. Therefore, the conservative Val to Leu mutation is likely benign. STAT6^Q633H^ is in the SH2 domain next to the phosphotyrosine binding site responsible for STAT dimerization (Figure 2C,D). The glutamine residue makes a hydrogen bond with Thr645, contributing to the stability of the phosphotyrosine-containing loop. The Q633H likely conserves this interaction, as histidine can form a similar hydrogen bond via its side chain. No drastic effects are therefore suspected from this mutation.

### 2.2. STAT5A and STAT6 Variants Are Associated with Enhanced Basal Immune Transcriptomes

**Figure 3:**
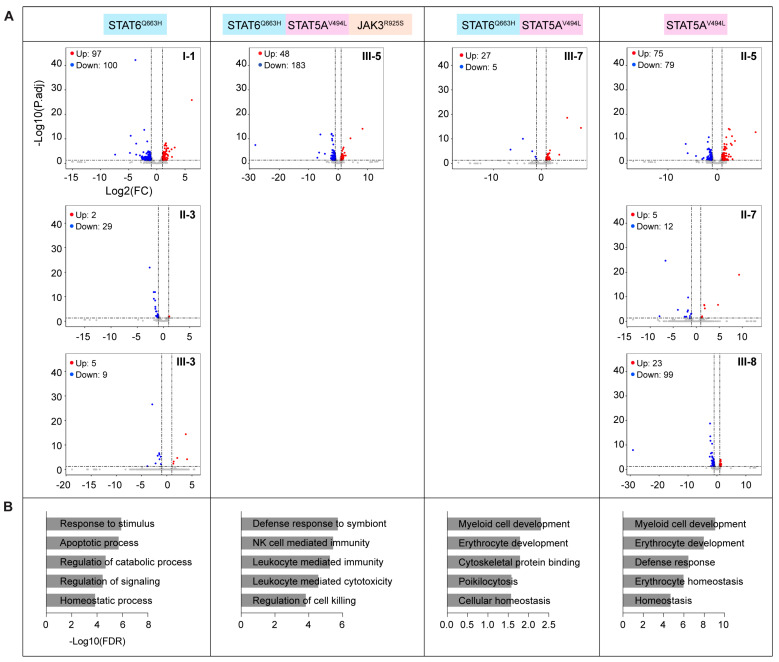
Impact of JAK/STAT variants on the baseline immune transcriptome in PBMC. (**A**) Volcano plots showing differentially expressed genes in PBMCs from the family members carrying distinct combinations of JAK/STAT variants. (**B**) Bar plots showing Gene Ontology (GO) terms enriched among significantly upregulated genes, along with their −log10(FDR) values.

To explore the potential impact of these *JAK*/*STAT* variants on the baseline immune transcriptome, i.e., in the absence of known infections, in a real-world setting, we conducted RNA-seq on peripheral blood mononuclear cells (PBMC) from 13 family members (Table S3). Specifically, we had baseline transcriptome data from five participants carrying the STAT6^Q633H^ variant. Three members (I-1, II-3, and III-3), spanning three generations carried only the STAT6^Q633H^ variant, while III-7 also carried the STAT5A^V494L^ variant. Individual III-5 carried both the STAT6^Q633H^ and the STAT5A^V494L^ variants as well as the JAK3^R925S^ variant. Volcano plots were generated to visualize differentially expressed genes in family members carrying JAK and/or STAT variants compared with family members without JAK/STAT variants (Figure 3A, Table S3). While a significant number of genes were deregulated in individual I-1 (STAT6^Q633H^ variant), her daughter and granddaughter did not display an aberrant immune transcriptome. The differences observed between these three individuals could be explained be age differences, with I-1 being 87 years old, II-3 55 years old, and III-3 36 years old. Alternatively, additional mutations in immune regulatory pathways could explain the result. Individual III-5 carrying the STAT6^Q633H^ variant in combination with the STAT5A^V494L^ and JAK3^R925S^ variants also presented an elevated transcriptome. Among the individuals carrying the STAT5A^V494L^, II-5 did display an enhanced transcriptome. Gene enrichment analysis of the significantly upregulated genes revealed activation of immune-related pathways, including homeostatic processes, immune response, and myeloid/erythroid cell development, in individuals harboring *JAK*/*STAT* variants (Figure 3B, Table S3). The presence of various combinations of [20] variants in these individuals (Table S1) complicates the interpretation of the findings.

**Figure 4:**
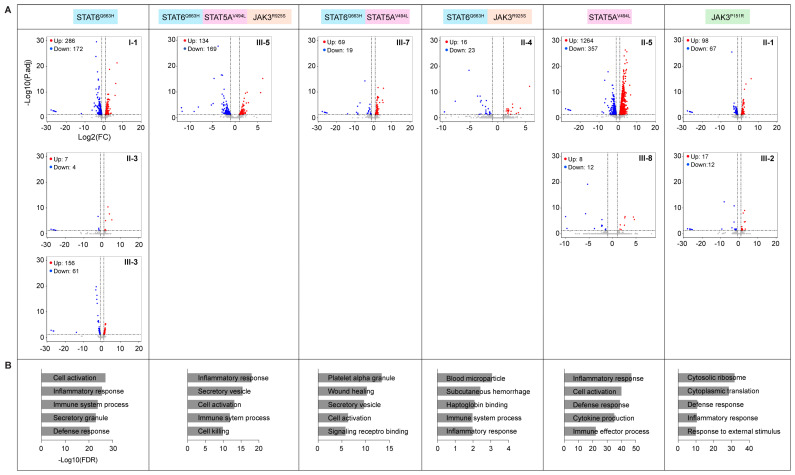
Effect of JAK/STAT variants on immune transcriptome of PBMC following Omicron infection. (**A**) Volcano plots of differentially expressed genes in PBMCs from family members two weeks after SARS-CoV-2 infection, stratified by JAK/STAT variant combinations. (**B**) Bar plots showing Gene Ontology (GO) terms enriched among significantly upregulated genes, along with their −log10(FDR) values.

We had previously analyzed RNA-seq data from a cohort infected with the SARS-CoV-2 Omicron strain [14], including 13 individuals of the family investigated in this study, and compared differentially expressed genes between individuals carrying JAK and/or STAT variants and family members without JAK/STAT variants. Blood had been collected approximately two weeks after the onset of symptoms and RNA-seq was conducted on PBMCs. Volcano plots demonstrated the highest induction of gene expression in I-1 (STAT6^Q633H^), II-5 (STAT5A^V494L^), III-7 (STAT6^Q633H^; STAT5A^V494L^), III-5 (STAT6^Q633H^; STAT5A^V494L^; JAK3^R925S^), and II-4 (STAT6^Q633H^; JAK3^R925S^) (Figure 4A, Table S4). Notably, individuals with elevated transcriptomes after SARS-CoV-2 Omicron infection also had elevated baseline transcriptomes (Figure 3), suggesting a genetic component, possibly the JAK/STAT variants described here. Across groups carrying distinct JAK and/or STAT variants, significantly upregulated genes were consistently enriched for inflammatory and immune response pathways compared with individuals lacking JAK/STAT mutations (Figure 4B, Table S4).

## 3. Discussion

The biological function of most germline and somatic human *JAK*/*STAT* variants remains elusive, and the impact of combinatorial SNVs and the possible role of epistasis on physiological responses in humans add additional layers of complexity. Such biological questions can be studied best in families carrying combinations of different variants. Here we identify four very rare *JAK*/*STAT* variants in a Tyrolean family with 17 members and integrate data-driven approaches and RNA transcriptomes with the goal to further elucidate their functions, alone and in combinations. We demonstrate that all four *JAK*/*STAT* variants are inherited in a Mendelian fashion without any evident clinical or physiological manifestations in the carriers. Most notably, only some carriers of specific variants presented with an altered immune transcriptome, suggesting a higher complexity of coinciding *JAK*/*STAT* and other immune regulatory SNVs.

With only limited clinical details available of the rare mutations, we employed in silico predictions, which, however, presented a complex and somewhat contradictory picture, with some tools suggesting potential pathogenicity while others indicated benign effects. While AI-driven AF3 is a very powerful tool to predict protein structures, it provided limited insight into the biological impact of the variants under investigation. The inconsistency among computational predictions highlights the difficulty of depending solely on in silico approaches to evaluate how mutations affect complex signaling pathways, emphasizing the importance of experimental confirmation. In vitro experiments, such as investigating JAK activities or chromatin binding of STATs, could provide limited information on individual variants; their combinatorial impact in individuals would be difficult to gauge.

Three variants, JAK3^P151R^, JAK3^R925S^, and STAT6^Q633H^ could be suspected as GOF variants because somatic versions are documented in the Catalog of Somatic Mutations in Cancer (COSMIC). The previous literature documented the presence of nine of the eleven known germline STAT6 GOF variants in COSMIC [13,25,26]. JAK3^R925S^ is a rare variant that has been reported in patients with T-ALL [27], but by itself, it does not transform Ba/F3 cells in vitro or stimulate cell proliferation of the MOHITO cell line [28]. While JAK3^R925S^ has been described as a passenger mutation in T-ALL patients, we suggest that it could impact the basal immune transcriptome and virus-induced innate immune responses in the presence of additional *JAK*/*STAT* mutation lines in proband III-5, who also carries the STAT5A^V494L^ and STAT6^Q633H^ variants. A possible synergy between *JAK* variants has also been reported in leukemia patients carrying two or more variants [29]. STAT6^Q633H^ is a very rare variant that has not yet been reported in the literature. However, one case has been reported in the COSMIC database, and PolyPhen2 reports it as pathogenic. This variant is located at the intersection of the SH2 and TAD domain, in close vicinity of well-known GOF variants, including P643R [13,26] and the key tyrosine phosphorylation site (Y641). Since only one out of the three individuals in our study carrying the STAT6^Q633H^ variant displayed an elevated basal and SARS-CoV-2-induced innate transcriptome, it is likely that additional variants in immune regulators contribute to its function. Like the STAT6 variant, only one of the three individuals carrying the very rare STAT5A^V494L^ displayed an enhanced basal transcriptome, again, suggesting the presence of variants in additional immune regulatory genes. Several hundred variants were identified in these individuals of other key immune genes, such as *TYK2* and interferon receptors, and the complexity of variant interactions remains to be investigated. The low AF of some of the mutations makes it unlikely that additional individuals carrying specific combinations of *JAK*/*STAT*/*TYK2* variants can be found. Only 1 out of 1 million individuals will carry two rare mutations, and some of our study participants carry up to four *JAK*/*STAT*/*TYK2* variants. This exemplifies the challenges in pinpointing the impact of variants in real-world settings.

Our findings highlight the combinatorial complexity of *JAK*/*STAT* variants in humans and their likely impact on physiology and pathophysiology. From a molecular and population genetics perspective, our findings are consistent with context-dependent associations compatible with incomplete penetrance and modifier-gene effects. It also underscores the importance of obtaining WGS data in any family study, permitting the identification of cooperating variants in related pathways. This presence of cooperating variants is highlighted by findings that the MPN driver mutation JAK2^V617F^ has been found in large cohorts of healthy elderly individuals [30]. Since mutations in humans do not exist in isolation, their impact can only be gauged in the context of additional mutations in their native setting. Understanding the complexity of epistatic SNP interactions has become a major challenge [21] in understanding how the co-presence of several variants can modulate physiological and pathophysiological responses. Artificial intelligence-driven algorithms will be critical in integrating the data on individual gene variants and their biological and clinical effects.

### Limitations

A limitation of the investigation is that we had identified only one extended family carrying the four JAK/STAT variants. The study depends on in silico predictions, structural modeling, and transcriptomic associations. These methods by themselves cannot determine gain- or loss-of-function effects. Functional in vitro assays could provide some biological information on individual JAK/STAT variants but would not yield significant information on the combinatorial impact of several co-inherited variants. An additional limitation is that the study spanned three generations but each generation contained only limited numbers of individuals to study, therefore we could not explore how age or sex might modify influence of a specific variant’s impact on the basal transcriptome.

## 4. Materials and Methods

### 4.1. Study Participants

Single-nucleotide variation (SNV) analyses were performed using 13 publicly available RNA-seq datasets derived from a previously described family infected with the SARS-CoV-2 Omicron variant [14,31]. Whole-exome sequencing (WES) data were available for nine of these individuals, along with six additional family members, yielding a total of 17 participants with SNV data (Figure 1). The cohort comprised nine females (mean age 49 ± 17 years) and eight males (mean age 45 ± 15 years), all of Austrian ethnicity and residing in Tyrol, Austria [14]. Owing to a limited sample size, the influence of sex and/or gender could not be assessed, which limits generalizability.

The study was approved by the Institutional Review Board (IRB) of the Office of Research Oversight/Regulatory Affairs, Medical University of Innsbruck, Austria (EK Nr: 1064/2021). All participants provided written informed consent. Data were coded and anonymized, and all procedures complied with relevant ethical guidelines and the Declaration of Helsinki (https://www.wma.net/policies-post/wma-declaration-of-helsinki-ethicalprinciples-for-medical-research-involving-human-subjects/, accessed on 1 June 2020).

### 4.2. Whole Exome Sequencing (WES) and Variant Calling

Genomic DNA was isolated from buffy coat samples using standard protocols (Wizard Genomic DNA Purification Kit, Promega, Madison, WI, USA). PCR-free libraries were prepared from 1 µg DNA (TruSeq DNA PCR-Free HT Sample Preparation Kit, Illumina, San Diego, CA, USA) and sequenced at the NIH Intramural Sequencing Center (NISC, Bethesda, MD, USA) on the NovaSeq 6000 and NovaSeqX+ platforms (Illumina), generating ≥300 million paired-end reads (2 × 151 bp) per sample.

Sequence alignment and variant calling were performed using the Clara Parabricks Pipeline (v4.0.1). Reads were aligned to the hg38 reference genome with BWA MEM [32], duplicates were marked using Picard [33], and base quality score recalibration (BQSR) and variant calling were conducted with GATK HaplotypeCaller, generating gVCF files with a minimum Phred-scaled confidence threshold of 16.

### 4.3. SNV Detection from RNA-Seq Data

RNA-seq reads derived from buffy coat samples underwent quality assessment with FastQC (v0.11.9) and adapter/quality trimming using Trimmomatic (v0.36) [34]. Reads were aligned using a two-pass STAR workflow (v2.7.9a) [35] to hg19, followed by duplicate marking with Picard (v2.9.2) [33]. SNV calling was performed using the GATK RNA-seq variant discovery workflow, including base recalibration with known polymorphic sites (dbSNP138) and variant calling with GATK HaplotypeCaller in discovery mode (ERC gVCF, minimum Phred score 30).

Variants were filtered using standard hard-filtering criteria (QD < 2.0 || QUAL < 30.0 || SOR > 3.0 || FS > 60.0 || MQ < 40.0 || MQRankSum < −12.5 || ReadPosRankSum < −8.0) and further refined by excluding repetitive and blacklisted genomic regions [36,37]. Only heterozygous or homozygous alternate SNVs (0/1 or 1/1) with read depth ≥ 10, allele frequency ≥ 10%, and without excessive coverage were retained [38,39,40,41]. SNVs near indels (±5 bp) were excluded as likely artifacts. Genomic coordinates were lifted from hg19 to hg38.

### 4.4. RNA Sequencing (RNA-Seq) and Data Analysis

Total RNA (1 mg) was depleted of ribosomal RNA, reverse-transcribed, and used for library preparation with the TruSeq Stranded Total RNA Library Prep Kit with Ribo-Zero Gold (Illumina, RS-122-2301). Paired-end sequencing (2 × 150 bp) was performed on a NovaSeq 6000 system (Illumina).

After quality control (FastQC (v0.11.9) and Trimmomatic (v0.36) [34]), reads were aligned to hg38 using STAR (v2.7.9a) [35]. Gene-level counts were obtained with HTSeq (v0.9.1) [42], normalized, and analyzed for differential expression using DESeq2 [43,44]. Confounding technical variation was addressed using RUVSeq [45]. Genes with fewer than ten total reads were excluded. Statistical significance was assessed using a paired, two-sided Wilcoxon test with Benjamini–Hochberg correction. Genes with log_2_ fold change >1 or <−1, pAdj < 0.05, and without a 0 value in any sample were considered significant and subjected to gene enrichment analysis (GSEA). Enrichment significance was evaluated using the Kolmogorov–Smirnov statistic.

### 4.5. Allele Frequency Annotation and In Silico Functional Prediction

Population allele frequencies were obtained from dbSNP, gnomAD, All of Us, COSMIC, and ClinVar databases. The potential functional impact of JAK/STAT variants was assessed using AlphaMissense [15], Rare Exome Variant Ensemble Learner (REVEL) [17], and PolyPhen-2 [16], applying default scoring schemes for pathogenicity prediction.

### 4.6. Structural Analyses

The monomeric JAK3 protein structure was predicted using AlphaFold3 (https://alphafoldserver.com) and visualized and analyzed using PyMOL (v3.1.6.1).

## 5. Conclusions

This study addresses the framework of the context-dependent impact of rare genetic variants in immune pathways. It demonstrates that four very rare germline *JAK*/*STAT* variants (JAK3^P151R^, JAK3^R925S^, STAT5A^V494L^, and STAT6^Q633H^) can be inherited across generations without overt clinical manifestations, yet selectively associate with enhanced basal and virus-induced immune transcriptomes in a subset of carriers. Structural and in silico analyses suggest largely modest individual effects, while transcriptomic data support that immune activation emerges in a context-dependent manner, likely driven by combinatorial and epistatic interactions with additional immune regulatory variants such as *TYK2*. These findings highlight that *JAK*/*STAT* variants function as genetic modifiers rather than standalone drivers of immune phenotypes and underscore the necessity of family-based, genome-wide, and functional approaches to understand how multiple rare variants collectively shape human immune responses.

## Figures and Tables

**Table 1:** *JAK* and *STAT* variants identified in study subjects.

Gene	AA Substitution	rs ID	gnomAD		All of Us		COSMIC	ClinVar	In Silico Pathogenicity Score		
			Allele Count	Allele Frequency	Allele Count	Allele Frequency	Case Number	Clinical Significance	Alpha Missense	Poly Phen2	REVEL
*JAK3*	P151R	rs55778349	12,489	7.8 × 10^−3^	5537	6.7 × 10^−3^	17	Benign	0.075	0.001	0.162
*JAK3*	R925S	rs149452625	1020	6.3 × 10^−4^	970	1.2 × 10^−3^	2	Not Reported in ClinVar	0.360	0.875	0.520
*STAT5A*	V494L	-	-	-	-	-	-	-	0.256	0.004	0.619
*STAT6*	Q633H	rs139828000	359	2.2 × 10^−4^	89	1.1 × 10^−4^	1	Not Reported in ClinVar	0.229	0.955	0.448
*TYK2*	A53T	rs55762744	14,980	9.3 × 10^−3^	6143	7.4 × 10^−3^	0	Benign	0.158	0.996	0.458
*TYK2*	V362F	rs2304256	446,002	2.8 × 10^−1^	200,826	2.4 × 10^−1^	31	Benign	0.083	0.015	0.053
*TYK2*	I684S	rs12720356	128,313	8.0 × 10^−2^	52,376	6.3 × 10^−2^	5	Benign	0.586	1.000	0.343
*TYK2*	P1104A	rs34536443	59,598	3.7 × 10^−2^	24,207	2.9 × 10^−2^	3	Benign/Likely benign	0.829	1.000	0.586

## Data Availability

RNA-seq generated in this study are available through GEO under accession number GSE313264. RNA-seq datasets from Omicron-infected individuals were obtained from GSE201530 [14] and GSE205244 [31]. WES data generated are not publicly available due to privacy constraints but may be accessed upon reasonable request to the authors.

## Supplementary Materials

The following supporting information can be downloaded at: https://www.mdpi.com/article/10.3390/ijms27020913/s1.

## Abbreviations

The following abbreviations are used in this manuscript:

JAK	Janus Kinase
STAT	Signal Transducers and Activators of Transcription
SNV	Single nucleotide variations
WES	Whole exome sequencing
