# Supplementary material for: Heritability and Transcriptional Impact of JAK3, STAT5A and STAT6 Variants in a Tyrolean Family
Source: Int J Mol Sci. 2026 Jan 16;27(2):913. doi: 10.3390/ijms27020913 (PMC12842485; doi:10.3390/ijms27020913)
Supplement: Supplementary file 1 [file ijms-27-00913-s001.zip › Supplementary Table S1.pdf]

Supplementary Table S1. Combinatorial occurrence of JAK/STAT variants in study cohort.

	JAK3		STAT5A	STAT6	TYK2			
ID	P151R	R925S	V494L	Q633H	A53T	V362F	I684S	P1104A
I-1								
II-1								
II-2								
II-3								
II-4								
II-5								
II-6								
II-7								
III-1								
III-2								
III-3								
III-4								
III-5								
III-6								
III-7								
III-8								
III-9								

Supplementary Table S2. Maximum-likelihood model scores for JAK/STAT and TYK2 variants identified across multiple diseases, generated using NeEDL (Epistasis Disease Atlas).

Supplementary Table S3. List of genes significantly regulated in the individual compared with the control group (family members without JAK/STAT variants). The list includes $\log_2$(fold change), p-values, adjusted p-values, and results from GSEA enrichment analysis.

Supplementary Table S4. List of genes significantly regulated in the individual compared with the control group (family members without JAK/STAT variants) at Day14 after Omicron infection. The list includes $\log_2$(fold change), p-values, adjusted p-values, and results from GSEA enrichment analysis.
